# *LGR5* expression and clinicopathological features of the invasive front in the fat infiltration area of pancreatic cancer

**DOI:** 10.1186/s13000-022-01203-w

**Published:** 2022-02-05

**Authors:** Masato Kamakura, Takeshi Uehara, Mai Iwaya, Shiho Asaka, Shota Kobayashi, Tomoyuki Nakajima, Yasuhiro Kinugawa, Tadanobu Nagaya, Takahiro Yoshizawa, Akira Shimizu, Hiroyoshi Ota, Takeji Umemura

**Affiliations:** 1 Department of Gastroenterology, Shinshu University School of Medicine, Matsumoto, Japan; 2 Department of Laboratory Medicine, Shinshu University School of Medicine, 3-1-1 Asahi, 390-8621 Matsumoto, Japan; 3 Department of Surgery, Shinshu University School of Medicine, Matsumoto, Japan; 4 Department of Biomedical Laboratory Medicine, Shinshu University School of Medicine, Matsumoto, Japan

**Keywords:** Leucine-rich repeat-containing G-protein-coupled receptor 5 (LGR5), RNA *in situ* hybridization, Pancreatic ductal adenocarcinoma, cancer stem cell, Fat invasion

## Abstract

**Background:**

Leucine-rich repeat-containing G-protein-coupled receptor 5 (LGR5) is a strong cancer stem cell marker in colorectal cancer; however, there are many unclear aspects of *LGR5* expression in pancreatic cancer. It has been reported that the interaction between tumor cells and stroma at the fat infiltration site has a significant effect on pancreatic cancer prognosis. Therefore, we report a clinicopathological study of *LGR5* expression at the fat invasion front in pancreatic cancer.

**Methods:**

*LGR5* expression was analyzed in 40 pancreatic ductal adenocarcinoma cases with RNAscope, which is a newly developed high-sensitivity *in situ* hybridization method. Epithelial-mesenchymal transition (EMT) was analyzed by the expression of E-cadherin and vimentin *via* immunohistochemistry.

**Results:**

*LGR5*-positive dots were identified in all cases, especially with glandular formation. In the fat invasion front, a high histological grade showed significantly reduced *LGR5* expression compared with a low histological grade (*p*=0.0126). *LGR5* expression was significantly higher in the non-EMT phenotype group than in EMT phenotype group (*p*=0.0003). Additionally, *LGR5* expression was significantly lower in cases with high vascular invasion than in those with low vascular invasion (*p*=0.0244).

**Conclusions:**

These findings suggest that decreased *LGR5* expression in the fat invasion front is associated with more aggressive biological behavior in pancreatic ductal adenocarcinoma, with higher tumor grade, EMT phenotype, and higher vascular invasion.

## Background

Pancreatic cancer (PC) is the seventh leading cause of cancer-related deaths worldwide [1]. The prognosis of PC patients is extremely poor. Early detection is important to improve the prognosis of PC. For early detection of PC, it is useful to understand the risk factors of PC such as family history, hereditary pancreatitis, hereditary diseases such as hereditary breast and ovarian cancer syndrome, underlying diseases such as diabetes, and lifestyle such as smoking. If PC is suspected by abdominal ultrasonography, computed tomography, magnetic resonance imaging, endoscopic ultrasonography, and endoscopic retrograde cholangiopancreatography are used for examination, with the aim of early detection. However, few PCs are diagnosed early, and only approximately 2% of all PCs are diagnosed at Stage 0 or I [2]. Recently, preoperative or postoperative chemotherapy have been performed in addition to surgery for PC, even in patients with Stage II or higher disease [3]. However, the 5-year survival rate is <10% [4].

Therefore, identifying factors that suggest prognosis in surgical materials is an important issue for PC. Most PCs are ductal adenocarcinomas (DAs) [5]. Analyzing the expression of various factors in DA or in the stroma surrounding DA may suggest important therapeutic targets. In this study, we focused on the expression of leucine-rich repeat-containing G-protein-coupled receptor 5 (*LGR5*) in DA. LGR5 was identified by lineage tracing to be a novel marker for adult stem cells in the small intestine, large intestine, and hair follicles [6] [7]. LGR5 is a seven transmembrane receptor [8] that is a target gene for Wnt/β-catenin signaling [7]. LGR5 is recognized as a cancer stem cell (CSC) marker for colorectal cancer [9]. The possibility of LGR5 being associated with CSCs has also been pointed out in pancreatic cancer [10] [11]. Wnt/β-catenin signaling is involved in various cellular functions including proliferation, migration, and drug resistance and is often dysregulated in cancer [12]. LGR5 is closely related to the control of Wnt/β-catenin signaling [13] [14]. Wnt/β-catenin signaling plays an important role in regulating the function of CSC [15]. *LGR5* has been shown to be a stem cell marker in previous studies including for gastrointestinal mucosa and gastrointestinal tumors, in which *LGR5* is the most promising stem cell marker. Subsequently, *LGR5* expression has been revealed in many organs and tumors. We have previously investigated *LGR5* expression in pancreatic ducts and DA [11]. Recently, greater attention has been paid to the fact that the microenvironment of the tumor infiltration area is distinct and related to the infiltration of tumor cells [16]. In PC, it has been suggested that the altered tumor microenvironment in surrounding adipose tissue, such as fatty acid release at the tumor infiltration front, may promote metastasis [17]. Additionally, it has been reported that fat invasion by tumor cells is associated with a worse prognosis [18]. Therefore, we focused on the expression of *LGR5* in the fat invasion front of DA and analyzed associations with clinicopathological features.

## Materials and methods

### Patients

We examined 52 cases of DA that were resected at Shinshu University between 2014 and 2019. Among them, eight cases with poor staining, one case with anaplastic carcinoma, and two cases without fat invasion were excluded. Stage II and III cases were also selected from the 52 total cases. Finally, 40 cases of DA with fat invasion were examined. We used the pancreatic tissue of a patient with extrahepatic cholangiocarcinoma for *LGR5* expression analysis, as well as normal pancreas. We obtained clinicopathological data including gender, age, histological grade (HG), vascular invasion, tumor infiltrating lymphocytes (TILs), lymph node metastasis, stage, and prognosis. Stage and histology were reconfirmed based on the 8th edition of the Union International Cancer Control TNM staging system and the 4th edition of the World Health Organization classification. Histology was also reassessed by two pathologists (T.U. and M.I.). The scores of TILs were measured in the fat invasion and assessed using a four-tier score as follows: none, 0; mild, 1; moderate, 2; and marked, 3 [19]. TIL was measured in the region in which *LGR5* expression was analyzed in one high-power field. Furthermore, TIL score was categorized as low-grade (score 0, 1, and 2) or high-grade (3).

This study was performed in accordance with the current ethical guidelines of the Declaration of Helsinki and was conducted in accordance with the requirements of the Institutional Review Board of Shinshu University School of Medicine (approval No. 4088).

### Histopathology and immunohistochemistry

We used surgically resected and formalin-fixed paraffin-embedded DA tissues. Optimal lesions with fat invasion were selected from hematoxylin and eosin (HE)-stained specimens. A tissue microarray (TMA) was then created by the procedure described below. Tissue cores were punched out from each donor tumor block using thin-walled 3-mm stainless steel needles (Azumaya Medical Instruments Inc., Tokyo, Japan), and cores were arrayed into a recipient paraffin block. Serial Sect. 4 μm in thickness were cut from these TMA blocks and stained with HE or immunostained with mouse monoclonal antibodies against E-cadherin (clone 36; dilution 1:2000; BD Biosciences, Franklin Lakes, NJ, USA) or vimentin (V9; dilution 1:50; Leica, Wetzlar, Germany). For antigen retrieval, sections were microwaved in 0.45% Tris/5 mM EDTA for 30 min. Detection of the primary antibodies was performed using an Envision detection system (Agilent Technologies, Santa Clara, CA, USA) according to the manufacturers’ recommendations. In accordance with a previous report [20], membranous E-cadherin expression was graded according to the proportion of positive cells and classified into four groups: 0, <10% of the cancer cells stained or with a complete absence of staining; 1, 10–49% positive expression; 2, 50–70% positive expression; and 3, >70% of cells with positive expression. Scores 0 and 1 were classified as E-cadherin negative, and scores 2 and 3 were classified as E-cadherin positive. For vimentin, clear positive staining in the cytoplasm of tumor cells was regarded as positive expression. We defined epithelial-mesenchymal transition (EMT) phenotypes into three groups according to the report by Aruga et al. [21]: non-EMT type, defined as E-cadherin positive and vimentin negative; incomplete EMT type, defined as E-cadherin negative and vimentin negative or E-cadherin positive and vimentin positive; and complete EMT type, defined as E-cadherin negative and vimentin positive. The incomplete EMT and complete EMT types were analyzed together as the EMT phenotype group and the non-EMT type was analyzed as the non-EMT phenotype group.

### *LGR5* RNA *in situ* hybridization

An RNAscope kit (Advanced Cell Diagnostics, Hayward, CA, USA) was used for *LGR5* mRNA expression analysis of TMA. RNAscope is a recently developed in situ hybridization technique with high sensitivity and low background. RNAscope uses a specific double “Z-shaped” probe to hybridize to target RNA sequences (approximately 18–25 bases). The probe then binds to amplifier probes that bind the chromogenic label (DAB). Briefly, tissue sections were pretreated by heating, and protease was applied prior to hybridization with the *LGR5*-specific probe. The detailed procedure was described in a previous publication [22]. The standard positive control (Mm-PPIB, ACD-313,902) and negative control (DapB, ACD-310,043) probes were used to ensure interpretable results. Brown punctate dots in the nucleus and/or cytoplasm indicated positive staining. *LGR5* expression was quantified under a 20× or 40x objective lens (Olympus BX51, Tokyo, Japan) according to the 5-grade scoring system recommended by the manufacturer (Table 1) [23]. Furthermore, *LGR5* mRNA expression was categorized into low expression (grades 0 and 1+) and high expression (grades 2+, 3+, and 4+). We selected one case from each score category, performed *LGR5* mRNA expression analysis in the whole section, and compared the scores in the fat invasion area. *LGR5* expression in PC was measured in the region where *LGR5* expression was strongest in the front of fat invasion. Additionally, the degree of cancer differentiation was also identified. Finally, we analyzed the relationship between *LGR5* expression and clinicopathological data and prognosis in DA patients, with particular regard to the overall survival (OS) rate.

**Table 1:** Interpretation of RNAscope amplification score according to the manufacturer’s guidelines. Staining scores 2, 3, and 4 were considered as high expression

Staining score	*LGR5* expression score
0, no staining or <1 dot every 10 cells, ×40 magnification	11
1+, 1–3 dots/cell, visible at ×20-40 magnification	11
2+, 4–9 dots/cell, very few dot clusters, visible at ×20–40 magnification	12
3+, 10–15 dots/cell, <10% of positive cells with dot clusters, visible at ×20–40 magnification	6
4+, >15 dots/cell, >10% of positive cells with dot clusters, visible at ×20 magnification	0

### Statistical analysis

Fisher’s exact test or Wilcoxon rank sum test were adopted to test for differences between patient subgroups. The survival rates of DA patients were calculated using the Kaplan–Meier method, and differences in those rates were compared by the Log-rank test. A *p*-value <0.05 was considered significant. All statistical analyses were performed using JMP Statistics software version 13 (JMP, Tokyo, Japan).

## Results

### *LGR5* expression and clinicopathological characteristics

**Fig. 1:**
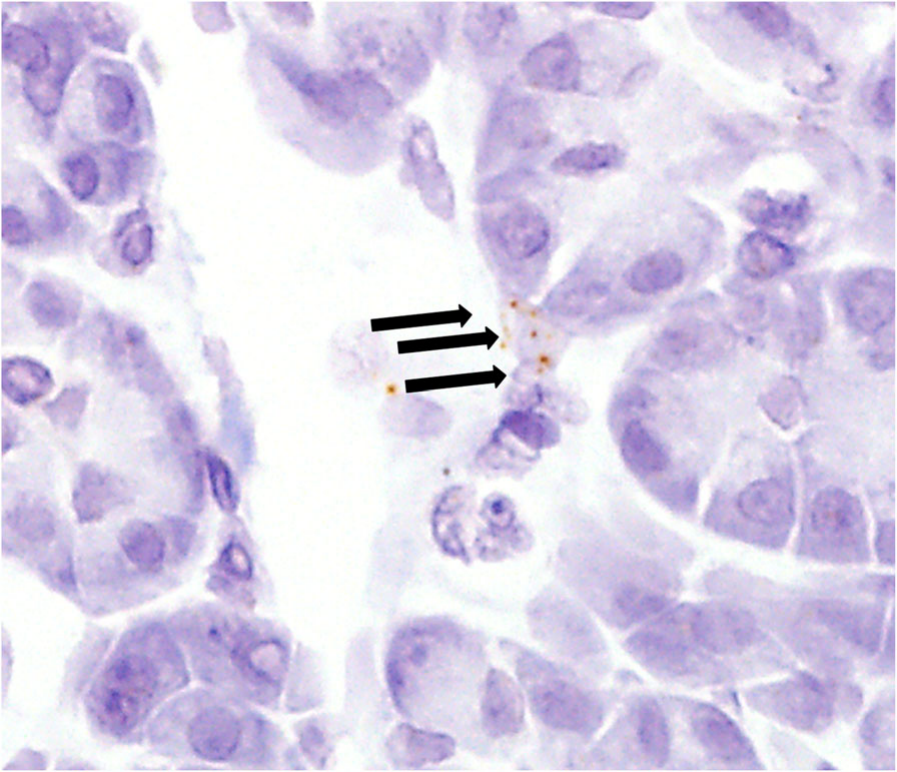
*LGR5* expression in normal pancreatic tissue. Positive dots were detected in intercalated ducts (arrows)

We first investigated *LGR5* expression in normal pancreatic tissue. *LGR5* staining was almost negative, but positive dots were detected in a very small number of intercalated ducts (Fig. 1).

**Fig. 2:**
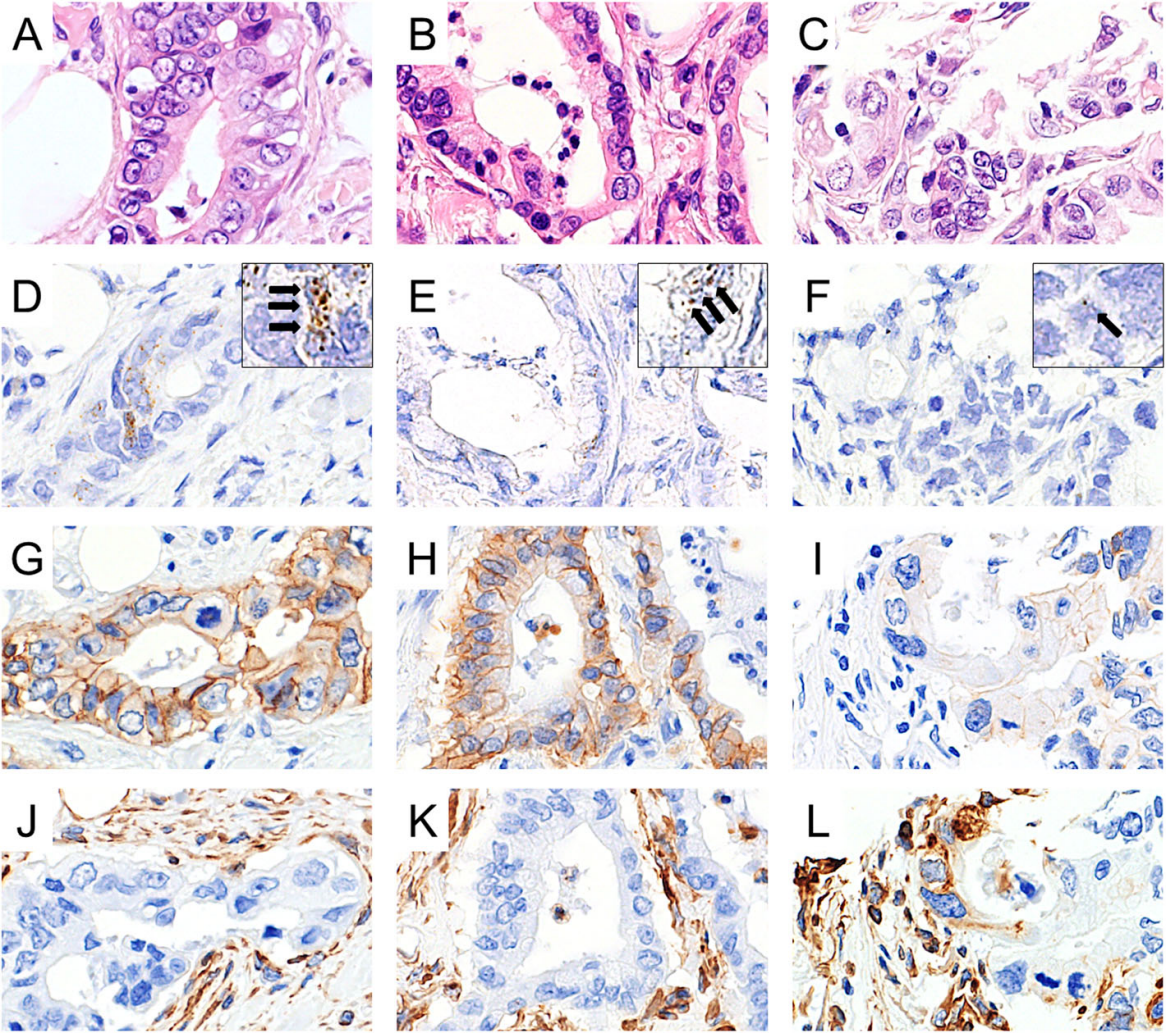
*LGR5* expression at the site of fat invasion. Representative hematoxylin and eosin (HE)-stained images of low histological grade (HG) (**A** and **B**) and high HG (**C**) tissues. In low HG tissues, *LGR5* expression was relatively easy to identify (**D** and **E**). Detailed images of *LGR5*-positive dots (arrows) are shown in the insert image in D and E. In high HG, low *LGR5* expression was identified (**F**). A detailed image of a *LGR5*-positive dot (arrow) is shown in the insert image in F. In low HG tissues, E-cadherin expression was identified (**G** and **H**). In high HG, E-cadherin expression was not identified (**I**). In low HG tissues, vimentin expression was not identified (**J** and **K**). In high HG, vimentin expression was identified (**L**). (A, B, and C: HE staining, 40× magnification; D, E, and F: *LGR5* RNAscope, 40× magnification [insert image 60× magnification]; G, H, and I: E-cadherin, 40× magnification; J, K, and L: vimentin, 40× magnification)

**Fig. 3:**
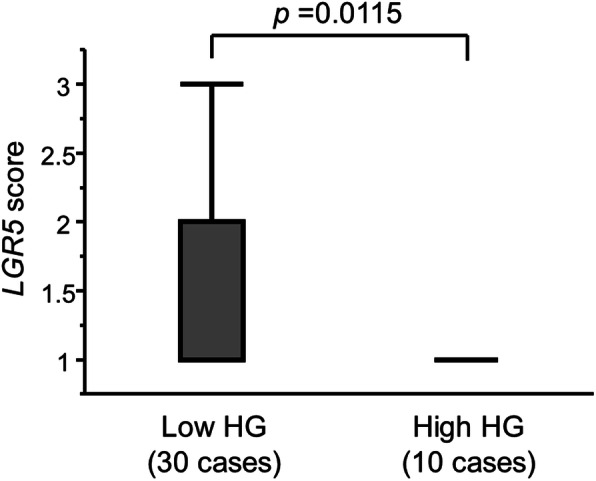
Associations between *LGR5* score and histological grade (HG). *LGR5* scores were significantly higher in the low HG group compared with in the high HG group (*p*=0.0115)

*LGR5*-positive dots were identified in all cases (Fig. 2). Although the localization of *LGR5*-expressing cells was uncharacteristic, many were identified in differentiating ducts. Regarding PC with fat invasion, there were 18 cases with high *LGR5* expression and 22 cases with low *LGR5* expression. Details are shown in Table 1. Relationships between clinicopathological variables and *LGR5* expression are shown in Table 2. *LGR5* expression was significantly lower at the site fat invasion in the high HG group than in the low HG group (*p*=0.0126). *LGR5* scores were also significantly higher in the low HG group compared with the high HG group (*p*=0.0115) (Fig. 3). Furthermore, *LGR5* expression was significantly lower in cases with high vascular invasion than in those with low vascular invasion (*p*=0.0244). *LGR5* expression was significantly higher in the non-EMT phenotype group than in the EMT phenotype group (*p*=0.0006). The *LGR5* expression score (score 1, 2, and 3) in fat invasion was consistent with TMA of the whole section.

**Table 2:** Associations between *LGR5* expression and clinicopathological characteristics in pancreatic ductal adenocarcinoma

		*LGR5* expression		
Factors	n	High (*n*=18)	Low (*n*=22)	*p*-value
Age				0.7512
≥69years		10	10	
<69 years		8	12	
Sex				1
Male		12	14	
Female		6	8	
Vascular invasion				**0.0244**
Present		6	16	
Absent		12	6	
TIL				0.4271
High		16	17	
Low		2	5	
Histological grade				**0.0126**
High		1	9	
Low		17	13	
EMT				**0.0006**
EMT phenotype		10	22	
Non-EMT phenotype		8	0	
TNM stage				0.4905
II		14	14	
III		4	8	

TIL: tumor-infiltrating lymphocyte; TNM: tumor, node, metastasis staging system

The HGs were as follows: 30 low grade cases (Grade 2: 30 cases) and 10 high grade cases (Grade 3: 10 cases). All patients were treated with adjuvant chemotherapy and no patients had neoadjuvant chemotherapy.

### Prognostic value of *LGR5* expression in pancreatic DA

**Fig. 4:**
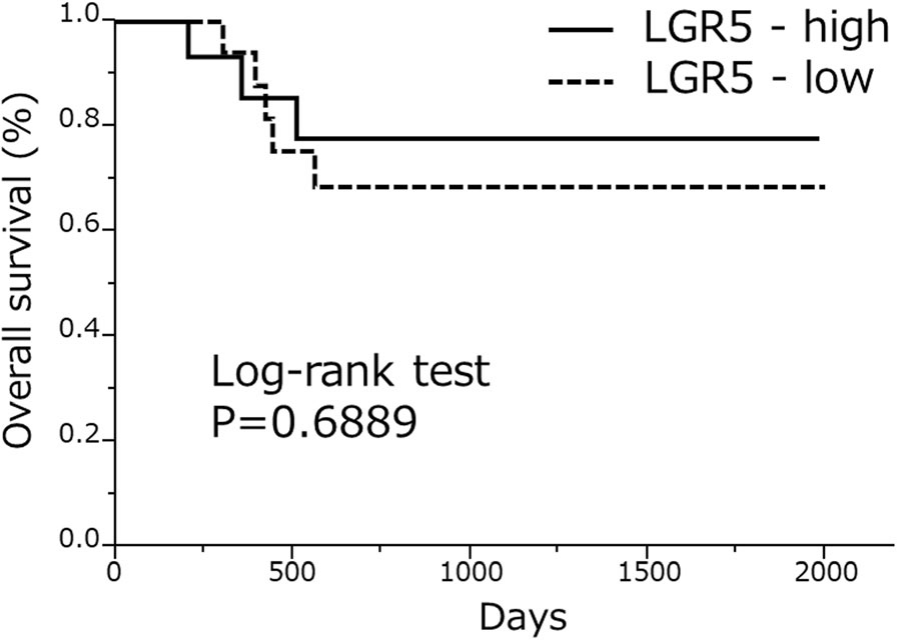
Prognostic value of *LGR5* by Kaplan–Meier analysis. There was no significant difference between overall survival (OS) in the high *LGR5* expression group compared with that in the low *LGR5* expression group (log-rank test *p*=0.6889)

Next, we assessed the prognostic value of *LGR5* expression in DA patients using Kaplan–Meier analysis and the log-rank test. The median survival of the entire DA patient group was 557.5 d (range: 293–1423 d). Log-rank analysis showed no significant difference between OS in the high *LGR5* expression group compared with in the low *LGR5* expression group (median OS: 696.5 d [range, 261–1524 d] vs. 549.5 d [range, 306.5–1091.5], respectively; *p*=0.6889) (Fig. 4).

## Discussion

Decreased expression of *LGR5* at the site of fat invasion in the high HG and EMT phenotype groups may suggest that *LGR5* affects the prognosis of DA, indicating that it may be related to cancer cell EMT. EMT is a change that induces the acquisition of migration and invasion abilities by epithelial-derived cancer cells and plays an important role in the multi-step process that ultimately ends with distant metastases [24]. Therefore, increased HG that suggests EMT of cancer cells, which has a significant effect on prognosis. Increased HG may also be biologically associated with low *LGR5* expression. It has been reported that *LGR5* is strongly expressed in differentiated adenocarcinomas [11], as well as in other carcinomas such as colorectal cancer [25] [26]. Low *LGR5* expression in high HG PC cases may have an important effect on prognosis, but further elucidation is warranted.

Low *LGR5* expression in the site of fat invasion may lead to EMT. In an immunostaining study of the colorectum, vascular invasion was frequently identified with low LGR5 expression [27], which is consistent with our study. Although the above trends differ from some previous papers [28] [29], there are some molecular biological reports that reinforce our view. Low *LGR5* expression may promote EMT, resulting in invasion and metastasis. Walker et al. reported that knocking down *LGR5* increased the activation of EMT genes and invasiveness of colorectal cancer cell lines [30]. Carmon et al. also reported that ablating *LGR5* resulted in decreased cell adhesion in colorectal cancer [31]. Jang et al. reported that *LGR5* expression was associated with favorable prognosis and that *LGR5* expression decreased migration in DLD1 cultured cells, which is one of the abilities gained by cells following EMT; however, they also found that *LGR5* expression enhanced migration in other cultured colorectal cells [32]. They also reported that EMT-related transcription factors were not involved in *LGR5*-regulated gene expression. These functional difference in colorectal cancer cells may indicate the complexity of the pathway that influences *LGR5* expression during EMT. Additionally, the pathway that controls *LGR5* expression during EMT may vary from organ to organ. Future elucidation is required.

Several reports have shown that both EMT and cancer stem cell markers are expressed in PC [33] [34]. It has been reported that *LGR5* and EMT-related transcription factors are co-expressed in intrahepatic cholangiocarcinoma [35], but re-verification of their *LGR5* expression data by immunohistochemistry and re-verification RNA *in situ* is desired. Jang et al. also reported no correlation between *LGR5* and the expression levels of other stem cell markers (CD133, CD44, CD24, and CD166) in colorectal cancer [32]. Therefore, *LGR5* may have different biological characteristics from other CSC markers.

It has been reported that decreased *LGR5* expression may be associated with abnormal methylation in colorectal cancer and bile duct cancer [32] [36]. In colorectal cancer, a relationship was highlighted between poor differentiation, lymph node metastasis, and low *LGR5* expression due to hypermethylation [32]. Conversely, distant metastasis and prognosis are associated with high *LGR5* expression due to hypomethylation [32], and the mechanism of methylation abnormalities has been identified. Methylation is closely related to microsatellite instability and may have effects on prognosis due to other factors. In this study, no significant correlation was found between *LGR5* expression and the amount of inflammatory cell infiltration, which may affect methylation.

The invasion of fat by cancer cells is known to secrete various adipokines such as leptin, adiponectin, IL-6, CCL2, and CCL5 [37] [38]. In cultured PC cells, it has been reported that invasion and drug resistance are enhanced in a fat invasion model [17]. It has also been reported that cancer-associated adipocytes transferred from peripancreatic adipocytes in the pancreas enhance tumor cell migration, invasion, chemotherapy resistance, and EMT properties [39]. First reported in breast cancer cells, cancer-associated adipocytes are known to be involved in the activation of adipokine CCL2 and lead to the further activation of cancer stem cells [40]. Because *LGR5* may have different properties than other cancer stem cell markers, there is an association between low *LGR5* expression in fat invasion and cancer-associated adipocytes, especially CCL2, which may result in EMT.

In normal pancreas, *LGR5* appears to be barely expressed or expressed in small amounts in some intercalated ducts. Our group previously reported similar results [11], indicating that *LGR5* expression may be enhanced during pancreatic regeneration. However, its function in the normal pancreas has not yet been fully elucidated.

## Conclusions

Although *LGR5* has been regarded as a promising cancer stem cell marker, its biological behavior may be different from other cancer stem cell markers; the exact function of *LGR5* remains unclear. The possibility of EMT occurring in cancer cells due to the involvement of *LGR5* and cancer-associated adipocytes needs to be reexamined by expression analysis in cultured cells. Our findings suggested that decreased *LGR5* expression in the fat invasion front is associated with more aggressive biological behavior in pancreatic ductal adenocarcinoma, with higher tumor grade, EMT phenotype, and higher vascular invasion. Further study is warranted in the future.

## Data Availability

All data generated and analyzed during the current study are available from the corresponding author upon reasonable request.

## Abbreviations

PC: pancreatic cancer
DA: ductal adenocarcinoma
LGR5: leucine-rich repeat-containing G-protein-coupled receptor 5
HE: hematoxylin and eosin
OS: overall survival
HG: histological grade
EMT: epithelial-mesenchymal transition
